# A novel fabrication method of copper–reduced graphene oxide composites with highly aligned reduced graphene oxide and highly anisotropic thermal conductivity

**DOI:** 10.1039/c9ra03743h

**Published:** 2019-06-07

**Authors:** Faisal Nazeer, Zhuang Ma, Yitong Xie, Lihong Gao, Abdul Malik, Muhammad Abubaker Khan, Fuchi Wang, Hezhang Li

**Affiliations:** School of Materials Science and Engineering, Beijing Institute of Technology Beijing 100081 China gaolihong@bit.edu.cn +86-10-68911144-865 +86-10-68911144-866; National Key Laboratory of Science and Technology on Materials under Shock and Impact Beijing 100081 China

## Abstract

Recently, metals with graphene and graphene oxide have been extensively used to enhance the mechanical and anisotropic thermal properties of composites. A novel facile fabrication approach of layer by layer self-assembly followed by hot press sintering was adopted to make copper–reduced graphene oxide composites. The microstructure and heat dissipation properties of pure copper and copper–reduced graphene oxide composites were analyzed with the help of SEM and continuous laser machine analysis. Thermal diffusivity of pure copper and copper–reduced graphene oxide composites was examined in different directions to measure the anisotropic thermal properties by using different volumetric percentages of reduced graphene oxide in the composites. Extraordinarily high anisotropic thermal conductivity of the copper–reduced graphene oxide composites was obtained at a very low concentration of 0.8 vol% reduced graphene oxide, with the difference between the thermal conductivity in-plane and through-plane being a factor of 8.82. Laser test results confirmed the highly anisotropic behavior of our copper–reduced graphene oxide composite with the remarkable property of heat dissipation. The three point bending test was also performed to check the flexural strength of the composites. At 0.6 vol% rGO, the flexural strength was noted (∼127 MPa), and it is 22% higher than that of pure sintered Cu. The high value of anisotropic thermal conductivity and higher flexural strength exhibited by the copper–reduced graphene oxide composite produced using a simple two-step fabrication method give us new hope to use these materials as heat sinks in thermal packaging systems.

## Abstract 2

Highly aligned rGO with anisotropic thermal conductivity was obtained in this work.

## Graphical abstract

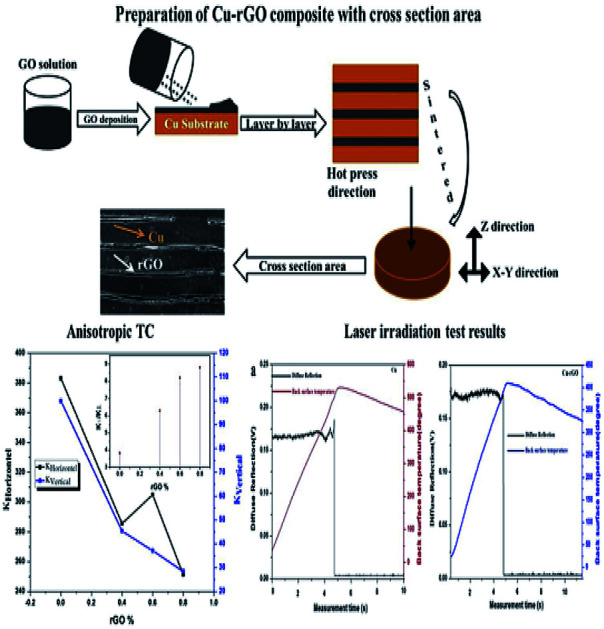

## 1. Introduction

Recent advancement in the miniaturization of electronic industry led to intensely dense packages, which causes an impressive increase in the amount of heat generated per unit volume. It is necessary to improve the miniaturization of electronic equipment for the advancement of heat dissipation technology. We need to make materials with properties that are properly designed and controlled, which fulfil the requirements of the heat dissipation industry. For this purpose, we would like to improve the anisotropic thermal properties of the materials that can be used in the field of thermal management systems as heat sinks. Most metals have good thermal dissipation properties compared with other materials (semi-conductors, polymers), so metals are the best choice for this purpose. Copper (Cu) has good values of thermal conductivity (TC), but its thermal conductivity values in different directions, *i.e.*, anisotropic thermal conductivity, differ little. To enhance the anisotropic thermal conductivity property of Cu, a few researchers used different fillers (reinforcement) such as graphite, graphene oxide and graphene.^1–15^

Graphene has extraordinarily high values of anisotropic thermal conductivity in-plane (4000–5000 W m^−1^ K^−1^)^16^ and through-plane (5–20 W m^−1^ K^−1^),^17^ so it is the best choice as a filler in a metal. Most of the researchers used graphene and its derivatives with metals and polymers.^17–23^ Boden *et al.*^13^ prepared Cu–graphene composites by using the powder metallurgy technique (ball milling + SPS). Their best sample showed an anisotropic thermal conductivity in-plane (292 W m^−1^ K^−1^) larger than through-plane by a factor of only 3. Some other researchers used pressure assisted techniques to improve the anisotropic thermal conductivity but, unfortunately, the results were frustrating. Fanyan Chen *et al.*^24^ adopted a (molecular level mixing + SPS) technique to align the graphene content within the Cu matrix. Even at 4 vol% graphene, the results of anisotropic thermal conductivity are not as good as expected. The difference between the in-plane and through-plane conductivity was a factor of 1.5. Graphene was not aligned properly by using these conventional methods of mixing. Cao *et al.*^25^ recently adopted a bio-inspired strategy to develop highly aligned graphene in the Cu matrix, and yield strength and elastic modulus increased significantly by 177% and 25% compared with pure Cu, respectively. However, they did not discuss the thermal properties of their composites.

We need to find an efficient way for the fabrication of copper–graphene composites with a high degree of alignment of graphene and to improve the anisotropic thermal conductivity of the composites. However, the study of graphene alignment in Cu composites is still rare.^13^ Most of the researchers used different techniques such as molecular level mixing,^26,27^*in situ* chemical vapour deposition,^28–30^ flake powder metallurgy,^26^ bioinspired preform impregnation^31^ and pulse reverse electrode.^32^ Unfortunately, they were not able to achieve a high value of anisotropic thermal conductivity even by using a very high concentration of fillers. Our previous results obtained by using the conventional method of powder metallurgy at a high percentage of 5 wt% rGO in a Cu–rGO composite showed a TC ratio difference of in-plane to through-plane of just 3.46. This result is not good enough, so we consider a layer by layer structure to enhance the ratio of TC. On the other hand, Cu–graphene films have also been examined, such as graphene–Cu–graphene heterogeneous film,^9^ RGO–graphene film^33^ and nitrogen doped graphene film.^34^ Although these films had high values of thermal conductivity and anisotropic thermal conductivity, there are many drawbacks of these composite films, such as they cannot be used on an industrial scale, because they can hardly be machined to the required parts with complex 3D shape limitations.

In this paper, we adopted an efficient route to achieve a high degree of alignment of reduced graphene oxide (rGO) within a copper (Cu) matrix by using a simple two-step fabrication method of layer by layer self-assembly followed by hot press sintering. It was found that the composites had highly aligned rGO and a well packed laminated structure, leading to an extraordinarily high anisotropic thermal conductivity. Laser irradiation test confirmed the highly anisotropic structure of the composites and enormous ability to dissipate the heat compared with pure Cu. This facile fabrication method and extraordinarily high values of anisotropic thermal conductivity may open up new doors for the development of Cu–rGO composites for thermal management applications.

## 2. Experimental

### 2.1. Synthesis of graphene oxide (GO)

Copper substrate of thickness 0.001 cm and natural graphite powder (purity > 99.99%, mesh size 1000 μm) were provided by Beijing Xing Rong Yuan Technology Co., LTD.

GO was synthesized by a modified hummer's method^35^ with a mesh size of 1000 μm of natural graphite flake powder. Initially, 25 ml (90%) of concentrated H_2_SO_4_ was heated at 90 °C, then 5 g of each of K_2_S_2_O_8_, P_2_O_5_ and 6.2 g graphite was added keeping temperature at 80 °C for 4.5 h. This solution was then treated with 200 ml of H_2_SO_4_ and 30 g of KMnO_4_ at a temperature <10 °C for 3 h under constant magnetic stirring. Finally, the solution was washed with 25 ml of H_2_O_2_ (30%) and HCL (5 wt%) 3 times.

### 2.2. Fabrication of Cu–rGO composites

**Fig. 1:**
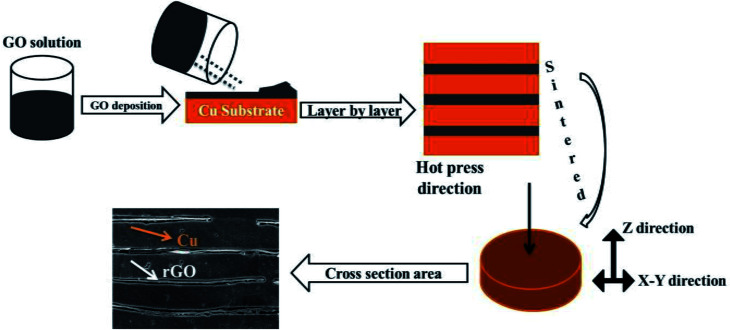
Schematic diagram of Cu–rGO composites formed by the layer by layer method.

Copper substrates were cut into pieces keeping the diameter of each substrate at 2.5 cm. Cu substrate pieces were placed on a square glass (*φ* = 24 × 24 cm) and GO (0.4, 0.6 and 0.8 vol%) was deposited on these Cu substrates by using ethyl alcohol as a solvent. GO deposited on Cu substrates was left at room temperature for 48 h for natural drying and ethyl alcohol evaporated. Cu–rGO composites formed in a layer by layer manner were sintered by using the hot press sintering machine for 3 h keeping the temperature at 950 °C and pressure at 35 MPa under an Ar^+^ gas atmosphere. During the sintering process, GO was reduced to rGO. Pure Cu substrates were also sintered under the same conditions for comparison. The whole process of composite preparation is illustrated in Fig. 1.

### 2.3. Characterization

The density of the bulk composites was measured by using the Archimedes principle.
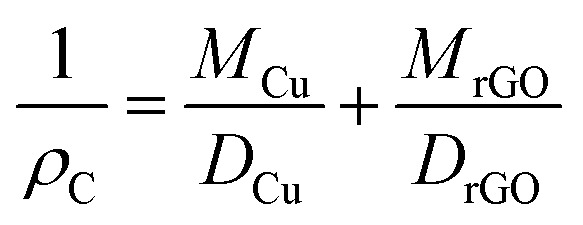
*ρ*_C_ is the density of the composite, *M*_Cu_ and *M*_rGO_ are the mass of copper and mass of reduced graphene oxide, while *D*_Cu_ and *D*_rGO_ are the density of copper and density of reduced graphene oxide, 8.96 g cm^−3^ and 2.25 g cm^−3^, respectively. Disorder in rGO was examined by using the Raman spectroscopy technique with an Ar^+^ laser wavelength of 532 nm (inVia-Reflex, Britain).

**Fig. 2:**
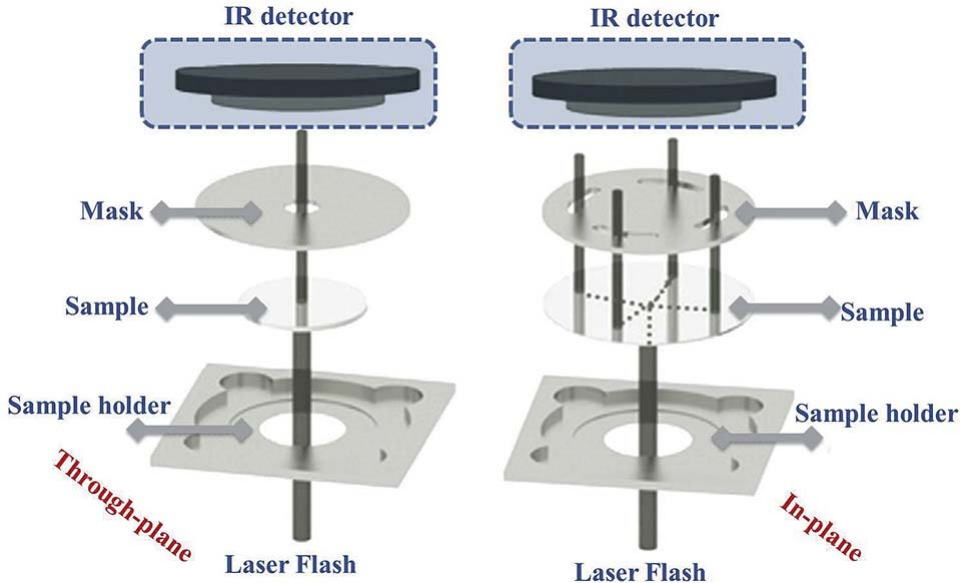
Schematic illustration of sample holders used for the measurement of the anisotropic thermal diffusion coefficient (*α*).

The microstructure of the bulk composite was investigated by using scanning electron microscopy (SEM, Hitachi S-4800, Japan). Thermal diffusivity of the composite was measured in both parallel and perpendicular directions by using a NETZSCH Laser Flash Analysis 467 machine at room temperature. The thermal conductivity of the composites was calculated by using the formula *K* = *αC*_p_*ρ*, where *α* is the thermal diffusivity and *C*_p_ is the specific heat capacity calculated by using the simple rule of a mixture of composites, 
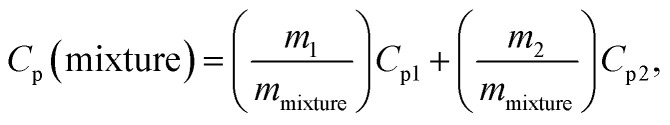
 where *C*_p_ is the heat capacity, *m*_1_ and *m*_2_ are the mass of Cu and rGO, *C*_p1_ and *C*_p2_ are the specific heat capacity of Cu and rGO, while *ρ* is the density of the composites. The same sample that have a thickness of 2 mm and diameter of 25 mm were used to determine the in-plane and through-plane thermal conductivity of pure Cu and the Cu–rGO composites, as shown in Fig. 2. Laser irradiation test was performed with a Nd:YAG continuous laser machine with 1070 nm wavelength by using laser power at 1000 W cm^−2^ and irradiation time was set to 5 s. The flexural strength of Cu and the Cu–rGO composites of dimensions 2 mm × 4 mm × 20 mm was measured by the three-point bending method with a crosshead speed of 0.5 mm min^−1^ and span of 30 mm at room temperature using an electromechanical universal testing machine (INSTRON-5566, Norwood, America). Three consecutive specimens were treated to gain average values of flexural strength.

## 3. Results and discussion

### 3.1. Raw powder and rGO characterization

**Fig. 3:**
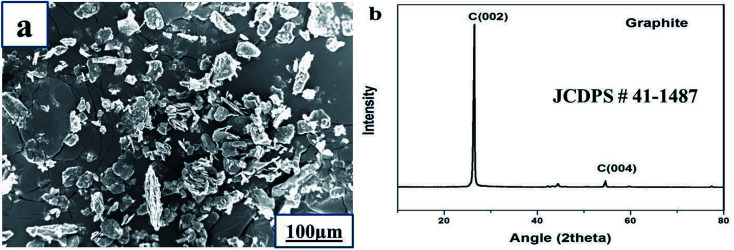
Graphite powder: (a) SEM image and (b) XRD pattern.

A SEM image of graphite powder is shown in Fig. 3a. The shape of the graphite powder particles is irregular. The XRD pattern of graphite powder is shown in Fig. 3b. There are two obvious peaks of graphite at an angle 2*θ* = 26.5° and 54.6° corresponding to the (002) and (004) crystal planes of the graphite flakes (JCDPS no. 41-1487), respectively. The *d*-spacing of the as-received graphite is 3.367 Å, which is approximately equal to the *d*-spacing of single crystal graphite, 3.353 Å. The narrow peaks and *d*-spacing values indicate that graphite has a highly graphitic nature, which is good for the thermal conductivity in the basal plane.^36^

**Fig. 4:**
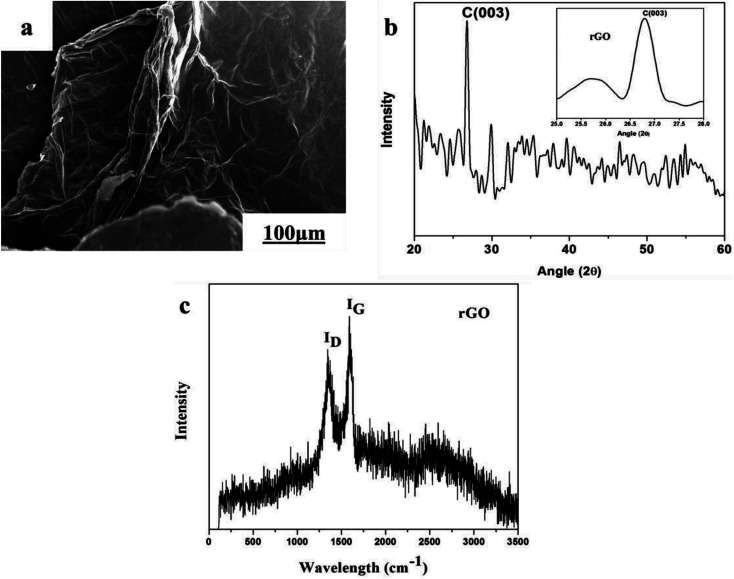
Reduced graphene oxide powder: (a) SEM image, (b) XRD pattern and (c) Raman analysis.

A SEM image and the XRD pattern of rGO powder are exhibited in Fig. 4. The morphology of rGO is flake-like, as shown in Fig. 3a. We observed a strong peak from rGO at an angle 2*θ* = 26.6° corresponding to the (003) crystal plane (JCDPS no. 26-1079). Mostly rGO peak signals were observed at the same angle, as shown in the inset of Fig. 4b. Raman analysis of the reduced graphene oxide powder shows the presence of *I*_D_ and *I*_G_ peaks in Fig. 4c. The typical D band value of carbon-based materials lies between 1280 cm^−1^ and 1450 cm^−1^ and the G band values are about 1580 cm^−1^. The D to G band ratio of graphene oxide is *I*_D_/*I*_G_ = 0.87. Higher values of D to G band ratio clearly indicate the presence of oxygen functional groups and broken aromatic rings.

### 3.2. Microstructure analysis of Cu–rGO composites

**Fig. 5:**
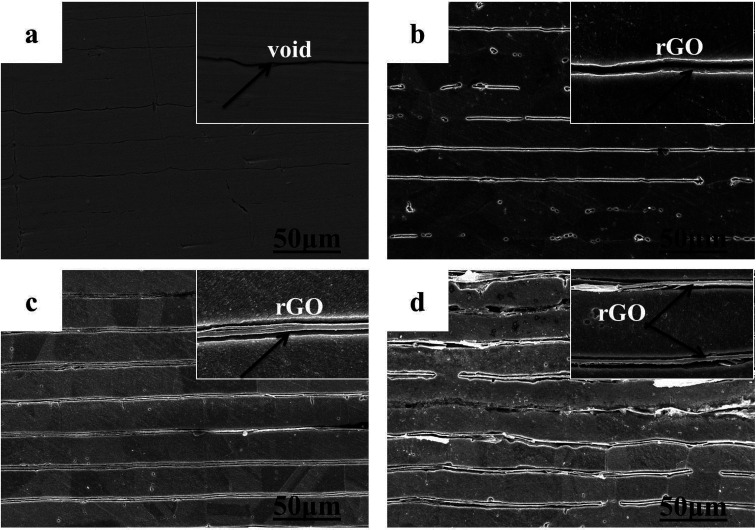
Cross-sectional SEM images of pure Cu and Cu–rGO composites (b–d): (a) pure Cu substrate and (b) 0.4 vol% rGO, (c) 0.6 vol% rGO, and (d) 0.8 vol% rGO (all inset figures show zoomed-in images).

Cross-sectional SEM images of the Cu–rGO composites are demonstrated in Fig. 5. Interfacial bonding between the Cu–rGO composites was not very strong and some voids are present, and the bonding inside rGO is also not strong enough and some voids were created and can be seen in Fig. 5. By increasing the amount of rGO within the Cu matrix, the size of the voids was enlarged, which can be seen clearly in the inset of Fig. 5b–d and is highlighted by the arrows, while the presence of rGO can also be observed clearly and is highlighted by arrows. This is due to the relatively big difference between the density of Cu and rGO and poor wettability property of the Cu matrix. The density of the Cu–rGO composites was observed to be lower due to the occurrence of these voids. At a low concentration of rGO, the voids are very few, but by increasing the amount of rGO, voids inside rGO and between Cu and rGO in the composites were seen clearly and the bending of rGO was also prominent. The presence of these voids may have a negative effect on the thermal properties of the composites. A SEM image of the pure Cu matrix after sintering can be seen in Fig. 5a for comparison with the Cu–rGO composites. The same problem occurred between the pure Cu matrix layers; interfacial bonding between two layers is not strong enough and some voids were clearly observed. Mostly, voids appeared between the interfaces of Cu and rGO. But, it can be seen from Fig. 5 that rGO is highly aligned and well-ordered within the Cu matrix with a little bending occurring due to the pressure applied during the sintering process. This will be beneficial for the improvement of anisotropic thermal conductivity.

### 3.3. High anisotropic thermal conductivity of Cu–rGO composites

**Fig. 6:**
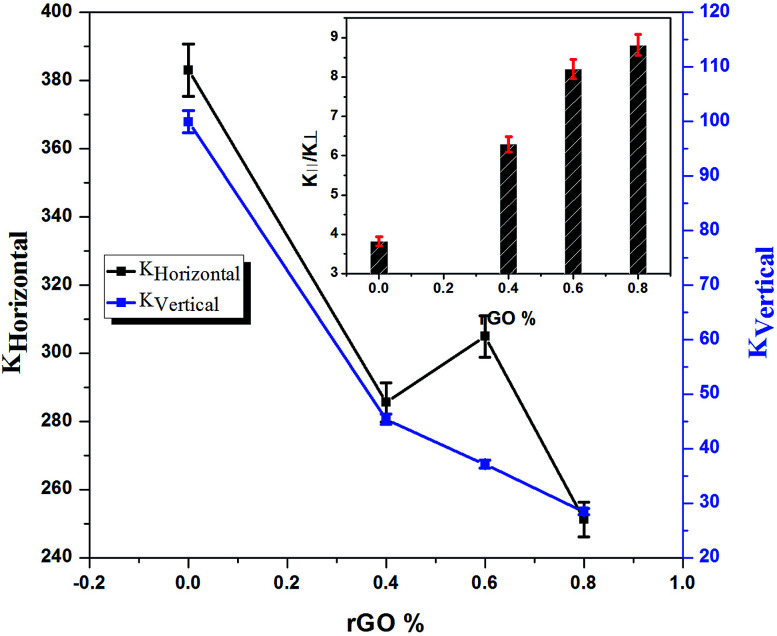
The anisotropic thermal conductivity of the Cu–rGO composites.

The thermal conductivity (TC) values of the composites in-plane (*K*_‖_) were 4–9 times greater than the through-plane (*K*_⊥_) values for different concentrations of rGO, due to the higher degree of alignment of rGO in the composites. By increasing the amount of rGO in the composites, the ratio between in-plane and through-plane TC was increased significantly. The maximum in-plane thermal conductivity for the Cu–rGO composites was observed when using 0.6 vol% rGO. The reason is that the density of 0.6 vol% Cu–rGO is 7.3644 g cm^−3^, which is relatively higher than that of the 0.4 and 0.8 vol% Cu–rGO composites, 6.3831 and 7.2825 g cm^−3^, respectively. However, the in-plane TC of Cu–rGO decreased upon increasing the amount of rGO and it was less than even pure Cu. The decreased value of TC is due to several reasons. Interfacial bonding between Cu and rGO was not strong and there are some voids present between them due to the poor wettability property of the Cu matrix, which causes the lower values of TC. Another reason is that there is some amount of oxygen also present in the interface region of the Cu–rGO composites, which produced lattice phonon dissipation. The presence of oxygen causes a decrease in thermal interfacial conductance and deterioration of the TC; a similar effect was observed for the functionalization of CNTs in CNT–Cu composites.^37^ Due to the large thermal expansion mismatch and poor adherence between Cu and rGO, the interfacial thermal resistance was raised. However, compared with other fillers used to form composites with Cu, the values of in-plane TC of the Cu–rGO composites are much better. In spite of this, the anisotropic TC ratio increased significantly (8.82) at a very low concentration of rGO (0.8 vol%) due to the higher degree of alignment of rGO, which was achieved by using a novel method for the fabrication of Cu–rGO composites, as shown in Fig. 6 (inset). Furthermore, rGO has a very high value of in-plane TC, hundreds of times better than that through-plane, and for the composites, with a low concentration of rGO, there is no literature available to the best of our knowledge to explain such a high difference between the in-plane and through-plane TC values. The fabrication method of the Cu–rGO composites used in this work is most suitable to get a higher anisotropic TC ratio.

### 3.4. Laser test

**Fig. 7:**
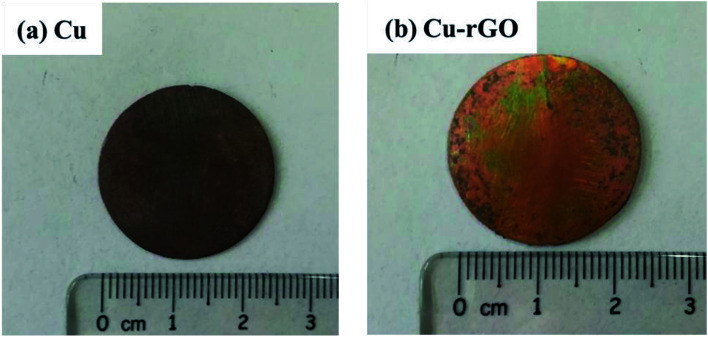
Samples after laser irradiation: (a) pure Cu and (b) Cu–rGO (0.8 vol% rGO).

**Fig. 8:**
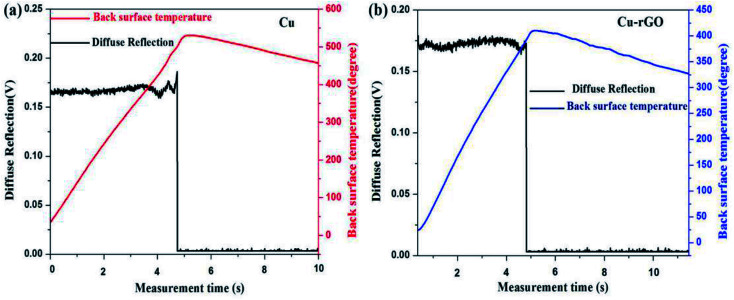
Laser irradiation test data. Laser power: 1000 W cm^−2^; irradiation time: 5 s. (a) Pure Cu. (b) Cu–rGO.

Laser test was done to verify the highly anisotropic structure of our best Cu–rGO composite (0.8 vol% rGO) sample and verify the heat dissipation property of the composite compared with pure Cu. Laser was irradiated on the pure Cu and Cu–rGO composite samples, as shown in Fig. 7. The power density of laser was set at 1000 W cm^−2^ and irradiation time was 5 s. After the laser was irradiated on the samples, we measured the back surface temperature of our samples with the help of a thermocouple. Our 0.8 vol% Cu–rGO composite sample exhibited a higher anisotropic thermal conductivity compared with pure Cu. An interesting result was obtained that the back surface temperature of the Cu–rGO composite was 120 °C less than that of pure Cu, as shown in Fig. 8, 29.3% lower than that of pure Cu. This is due to the large ratio between (horizontal) in-plane (251.23 W m^−1^ K^−1^) and (vertical) through-plane (28.48 W m^−1^ K^−1^) TC for the Cu–rGO composite, 8.82, and for Cu, the ratio was 3. This means that the large difference of anisotropic thermal conductivity values of Cu–rGO plays an important role in heat dissipation. rGO was also highly aligned within the composite, which enables good heat dissipation, and the back surface temperature of the Cu–rGO composite after laser irradiation was lower compared with pure Cu.

### 3.5. Flexural strength of Cu–rGO composites

**Fig. 9:**
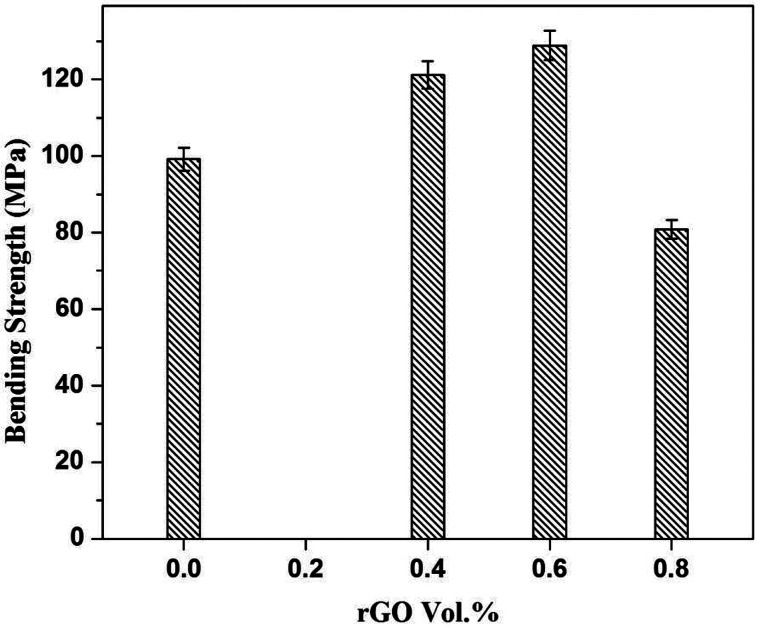
Flexural strength of pure Cu and Cu–rGO composites.

The three point bending test was performed to investigate the flexural strength of pure sintered Cu and the Cu–rGO composites, as shown in Fig. 9. The flexural strength of pure Cu was observed (∼99 MPa), while adding the rGO into the Cu matrix caused the flexural strength to firstly increase significantly and then decrease with increasing the concentration of rGO in the composites. At 0.4 and 0.6 vol% rGO in the composites, the flexural strength was ascertained (∼121 and 127 MPa), being 18% and 22% higher than pure Cu, respectively. With a low concentration of rGO, the interfacial bonding between Cu and rGO is better than at a higher concentration of rGO, and the densities of the composites also show significant changes. At a higher concentration of 0.8 vol%, the density of the composite is slightly lower compared with other composites due to the presence of voids, which also lead to a decrease in the flexural strength of the composites. The voids decrease the density as well as the mechanical properties of the composites, as has happened in our previous work.

## 4. Conclusions

In summary, Cu–rGO composites were successfully fabricated by a simple and novel two step method of layer by layer self-assembly followed by hot press sintering. The most important advantage of this method is that rGO was highly aligned within the Cu matrix, while interfacial bonding between Cu and rGO was not so strong and voids can be seen in the interfacial area. But, due to the highly aligned rGO, we obtained highly anisotropic thermal conductivity values of the Cu–rGO composites. Thermal conductivity in-plane (251.23 W m^−1^ K^−1^) and through-plane (28.48 W m^−1^ K^−1^) show the highest difference with a ratio of 8.82. Laser irradiation tests also confirmed the good alignment of rGO in the Cu matrix and highly anisotropic behavior of the Cu–rGO composites with a greater heat dissipation property. The value of back surface temperature (330 °C) of the Cu–rGO composite (0.8 vol% rGO) was 29.3% less than that of pure Cu (450 °C). The flexural strength of the Cu–rGO composites was also significantly increased compared with pure Cu. At 0.6 vol%, the flexural strength was ascertained to be ∼127 MPa, which is 22% higher than that of pure Cu. A simple two-step facile fabrication method, involving highly aligned rGO and highly anisotropic thermal conductivity values with such a low percentage of rGO, was reported; this has not been observed before in the literature, to the best of our knowledge, to date. These composites may be used as heat sinks in thermal packaging systems and the electronics industry.

## Conflicts of interest

There are no conflicts to declare.

## Supplementary Material
